# A new genus of Neelidae (Collembola) from Mexican caves

**DOI:** 10.3897/zookeys.569.5984

**Published:** 2016-02-24

**Authors:** Vladimír Papáč, José G. Palacios-Vargas

**Affiliations:** 1 State Nature Conservancy of the Slovak Republic, Slovak Caves Administration, Cave Care Department, Železničná 31, 979 01 Rimavská Sobota, Slovakia; 2 Laboratorio de Ecologia y Sistemática de Microartrópodos, Departamento de Biologia, Facultad de Ciencias, Universidad Nacional Autónoma de México, 045 10 México, D.F.

**Keywords:** Neelidae, Taxonomy, Mexico

## Abstract

The new genus *Spinaethorax*, whose proposal is based on specimens of *Megalothorax spinotricosus* Palacios-Vargas & Sánchez, 1999, is given a new name combination and a redescription. The type species comes from two caves in Campeche State, México. A new combination is also suggested for *Megalothorax tonoius* Palacios-Vargas & Sánchez, 1999. The new genus is similar to *Megalothorax* Willem, 1900 and *Neelus* Folsom, 1896, but it clearly differs from all genera within family Neelidae by a peculiar combination of characters and the presence of some new features, e.g. globular sensillum on Ant. III, sword-like macrosetae on oral fold. A comparative table and an identification key for all Neelidae genera as well as some summary tables of antennae chaetotaxy and legs setation for type species are provided.

## Introduction

The family Neelidae comprises five genera and 41 species in the world. However, only 9 species in 3 genera are known from Mexico (Palacios-Vargas 1997; Palacios-Vargas and Sánchez 1999). This family is mainly a euedaphic group of Collembola, whose members are usually very small (0.3–1.0 mm), they have nor eyes or scales, however, their antennae are shorter than their heads. The main differences among the genera of this family are in the structure of forehead setation, antennae, sensory fields and furcula.

Members of this family are cosmopolitan. Therefore, they have been found in various localities mainly associated with soil and litter at different altitudes from sea shore up to 3,000 m a.s.l. (García-Gómez et al. 2009). They have frequently been recorded from caves, mainly in places with rich organic material. This family has two endemic genera: *Acanthoneelidus*, with only one species from Europe, and *Zelandothorax* from New Zeland plus three cosmopolitan genera. *Megalothorax* is the most diversified with 28 species, *Neelus* with 6 species *Neelides* with 5, both widely spread, too. The new genus described herein has two species distributed in Mexican caves.

The most remarkable contribution on this family from Mexico is the work by Bonet (1947), who revised the whole family of Neelidae. The catalog by Palacios-Vargas (1997) records 7 species in this family but some of them have to be revised in the light of new characters. The most recent contribution to the taxonomy of this family from Mexico was that by Palacios-Vargas and Sánchez (1999) who described the two new species that are revised herein.

## Materials and methods

The present redescription of *Megalothorax spinotricosus* Palacios-Vargas & Sánchez, 1999, is based on original slides deposited in Facultad de Ciencias, UNAM. Specimens were obtained from samples of bat guano and soil that were processed by Berlese-Tullgren funnels and preserved in 75% ethylalcohol. Slides were mounted using Hoyer´s solution.

Body length was measured on slides excluding antennae and furcula. Lengths of unguis and unguiculus were measured between the most basal (proximal) point and the tip on their inner margins. Besides the common measurements, the ratio “unguis I, II, III (inner margin): Ti. I, II, III width (middle part)” that can be used as additional character, was also included.

We followed nomenclature used in last *Neelus* revision (Kováč and Papáč 2010) for labral setae and dental spines. Nomenclature used in the most recent revision of *Megalothorax* after Schneider and D´Haese (2013) was applied for arrangement of sensory fields, wax rod crypt (wrc1–8) on head, Th. and Abd. sensilla s1, s2, s3, subsegments of dens and chaetotaxy of antennae. Forehead chaetotaxy (presence of a0 seta) is applied according to Deharveng (1978) and posterior chaetotaxy of head is used according to Palacios-Vargas and Sánchez (1999).

Abbreviations: Ant.—antennal segment; Th.—thoracic segment; Abd.—abdominal segment; Ti.—tibiotarsus; scx—subcoxae; s.f.—sensory field; wrc—free wax rod generating crypt; s1, s2, s3, s3´—swollen sensilla; dp—proximal part of dens; dd—distal part of dens; UNAM—Universidad Nacional Autónoma de México.

## Taxonomy

### Spinaethorax gen. n.

Taxon classificationAnimaliaCollembolaNeelidae

http://zoobank.org/9E27A3C3-9464-4A6A-94B8-5BB470502D2C

#### Diagnosis.

A genus of the Neelidae Folsom, 1896 with the following diagnostic characters:

Habitus of Neelidae. Small size, about 0.6 mm. Color white. Tegumentary grain fine and uniform. Apex of head with sword-like spines, body with several such spines, mainly around sensorial fields. Ant. III and IV fused and Ant. III with small globular sensillum in proximal position. Anterior labral setae R_1_ and R_2_ thick, curved and smooth. Oral fold with 1+1 sword-like macrosetae. Basomedian field of labium furnished with 6+6 setae. Presence of 3 setae around abdominal sensory fields, no E3 spine/setae on dd. Mid abdomen with swollen sensilla s3 and s3´. Base of Abd. IV sternite with 1+1 neosminthuroid setae, smooth and with pointed tip.

#### Type species.

*Spinaethorax spinotricosus* (Palacios-Vargas & Sánchez, 1999), comb. n.

#### Redescription.

Figs 1–15.

**Figures 1–5.**
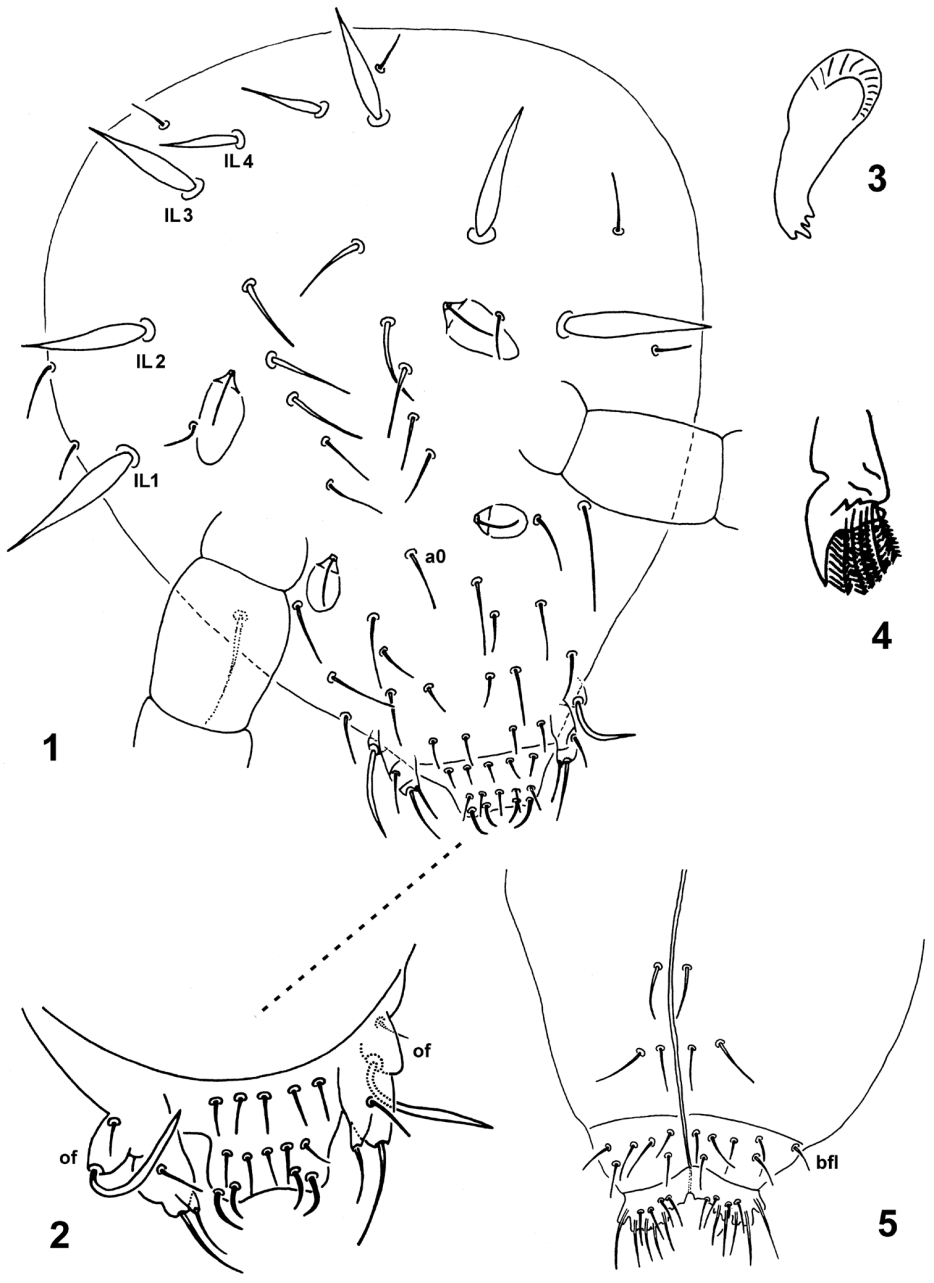
*Spinaethorax spinotricosus*. **1** dorsal chaetotaxy of head **2** anterior part of head with labrum, of—oral fold **3** mandible **4** maxilla **5** labium with ventral head back, bfl—basolateral field of labium.

**Figures 6–10.**
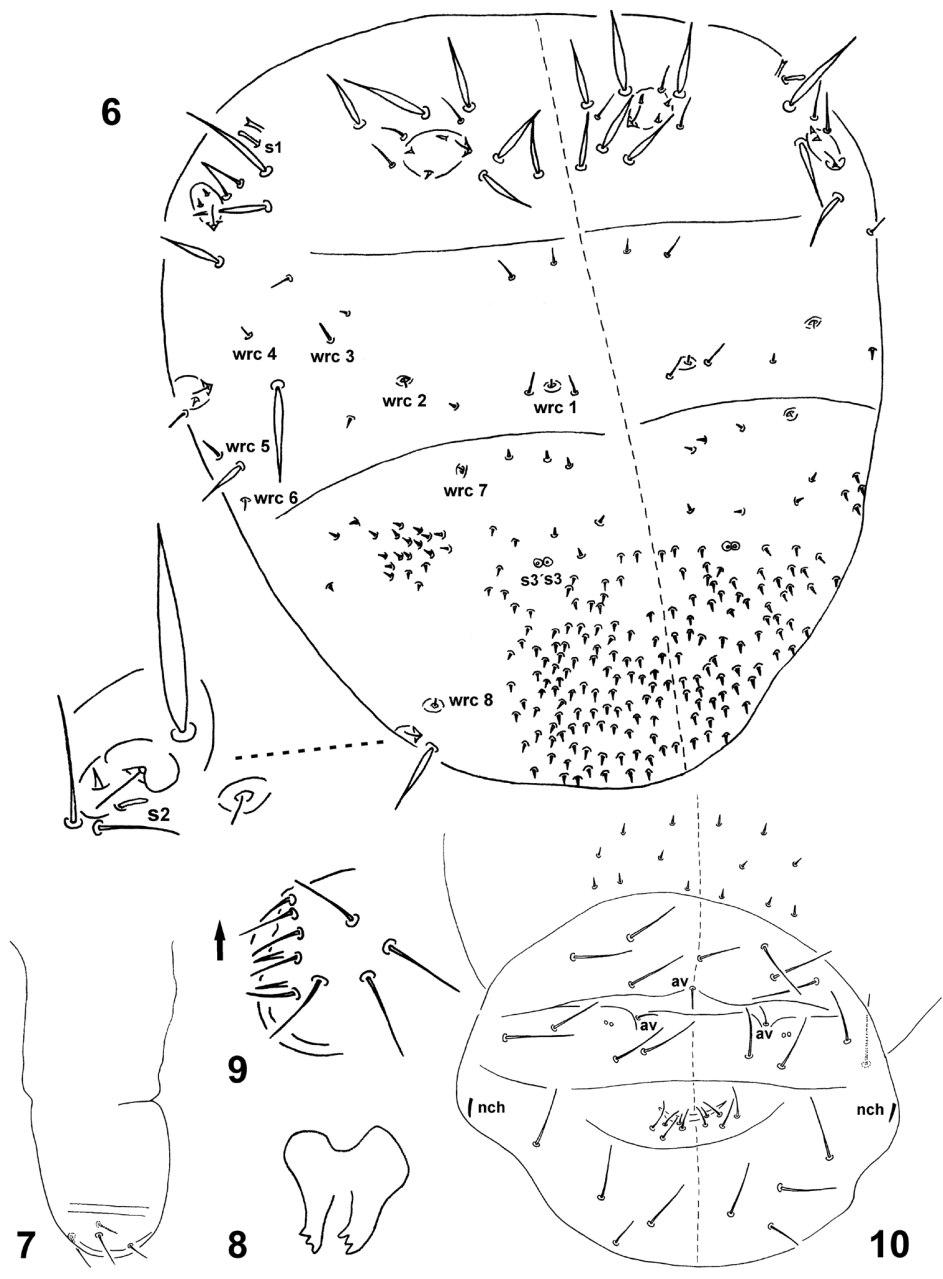
*Spinaethorax spinotricosus*. **6** thoracic and abdominal chaetotaxy with abdominal sensory field enlarged **7** ventral tube in lateral view **8** tenaculum **9** male genital plate in lateral view, arrow shows anterior direction **10** female genital plate frontal view, av—anal valve setae, nch—neosminthuroid setae.

**Figures 11–12.**
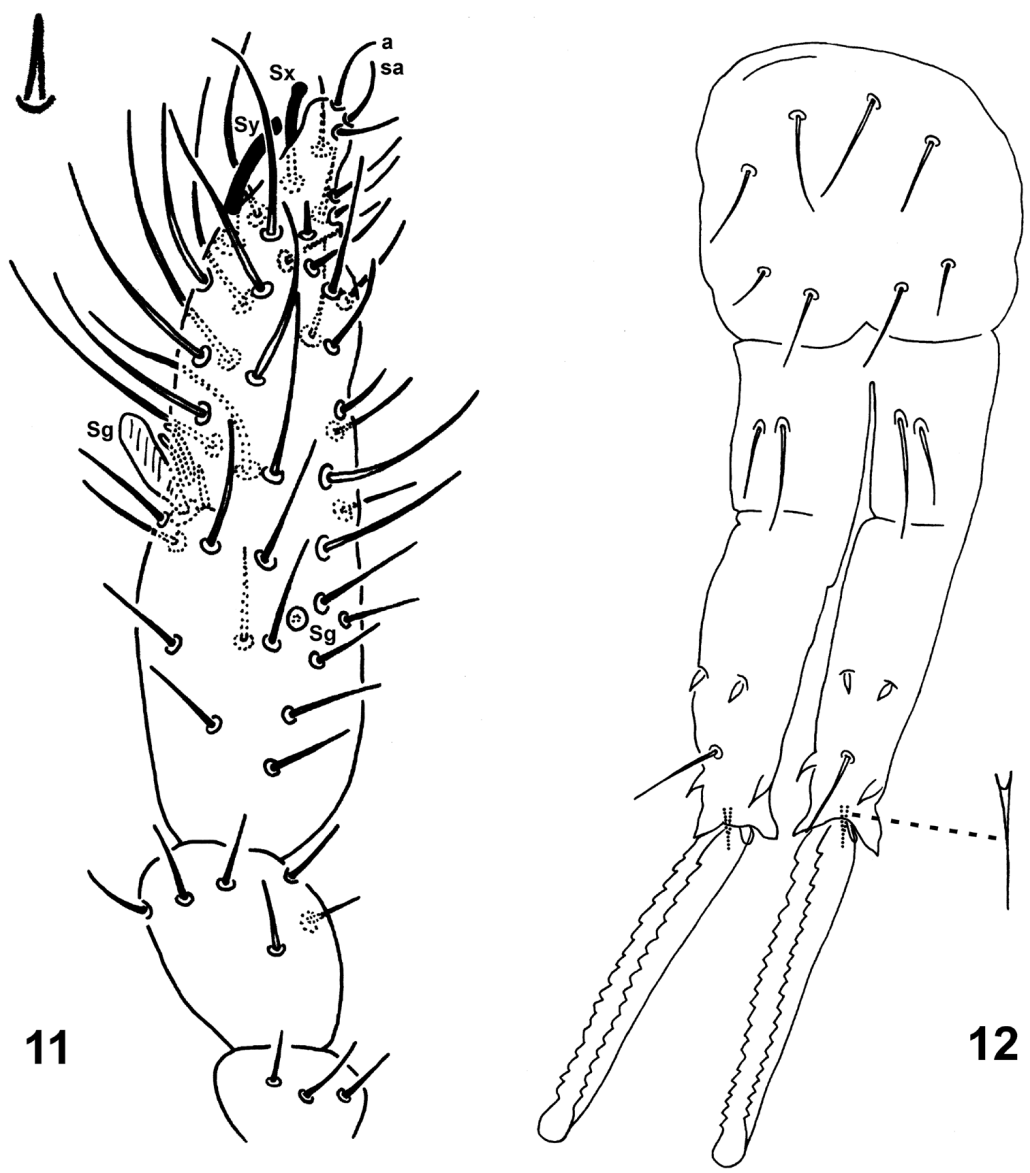
*Spinaethorax spinotricosus*. **11** dorsal Ant. I–IV, with Ant. IV organ enlarged **12** furcula, posterior view with anterior seta enlarged.

**Figures 13–15.**
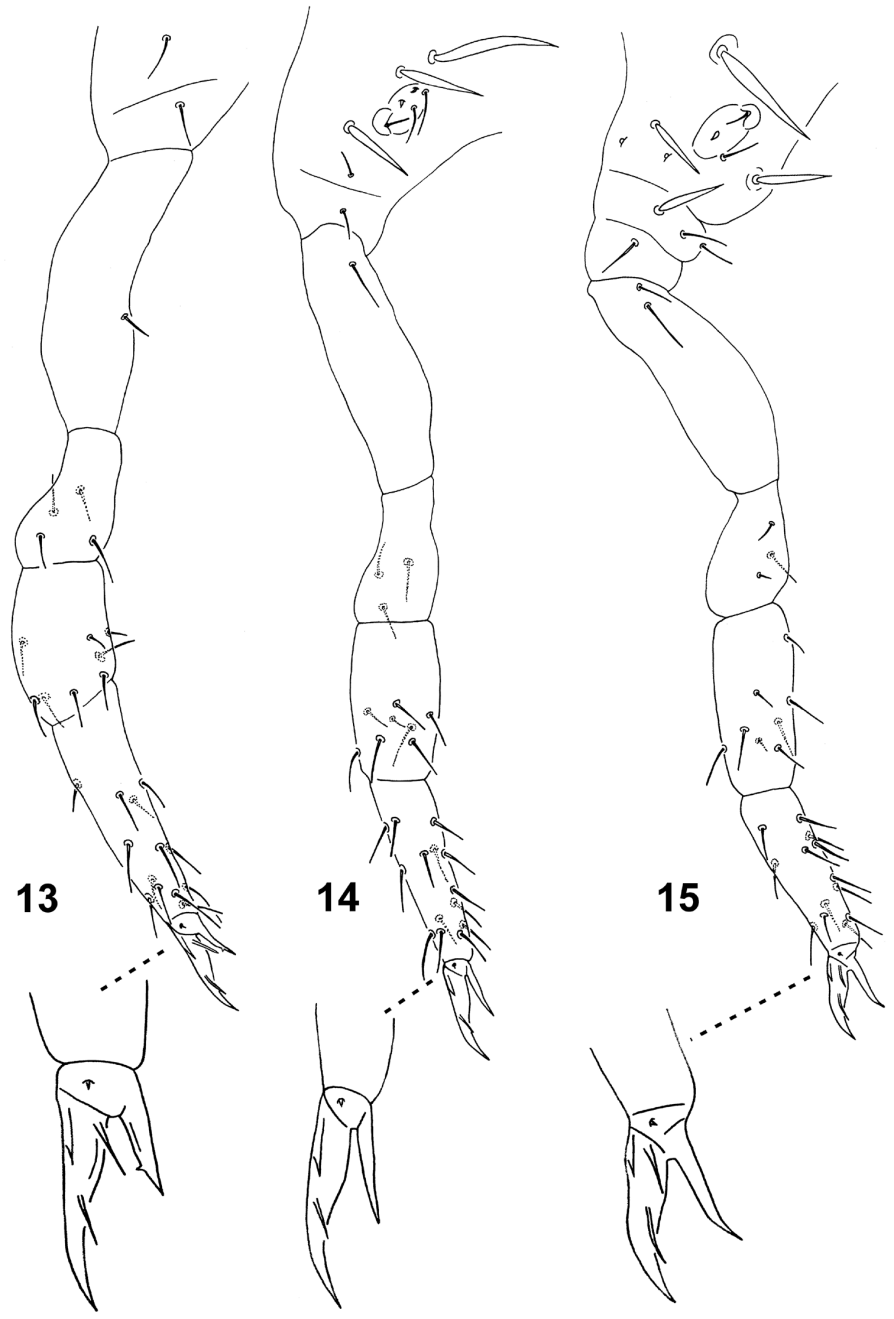
*Spinaethorax spinotricosus*. **13** leg I **14** leg II **15** leg III, with detail of each feet complex.

#### Type material.

Holotype: female mounted on slide. Original label: 23/00/1991, Mexico, Campeche, Cueva Xtancumbilxunaan (cave), 29.xii.1996, A. Ruíz and S. Aguilar col., ext. soil. Paratypes: 1 female on slide, 23.viii. 1991, J. G. Palacios col., direct collection and 2 juveniles on slides, the same data as the holotype. Type material deposited at Facultad de Ciencias, UNAM.

#### Other material.

Mexico, Campeche, Cueva Actún Guachapil (cave), 1 male, 1 female and 2 juveniles on slides. Original label: 14/iv/2012, 22.iii.1997, A. Ruiz and S. Aguilar col., ext. guano; 1 female on slide, 29.v. 1997, J. G. Palacios col., ext. guano. Material deposited at Facultad de Ciencias, UNAM.

#### Diagnosis.

Unpaired seta a0 between antennal basis. Labral chaetae R_1_ and R_2_ thick, curved and smooth. Oral fold with 1+1 sword-like macrosetae. Basomedian field of labium with 6+6 setae. Ant. III with small globular sensillum. Manubrium with 4+4 posterior setae. Mucro with both lamellae serrated and with rounded tip.

#### Description.

For lengths of different character see Table 1. Body length 0.5–0.7 mm. Habitus globular, as other members of Neelidae. No pigmentation, cuticle finely granulated, *linea ventralis* without crossing with integumentary channels on ventral head back. Mid and hind Abd. with numerous spine-like microsetae, stouter spines around all sensory fields on body and apex of head.

**Table 1.** Lengths (µm, mean in parenthesis) of different morphological characters of *Spinaethorax spinotricosus* comb. n. (male, females and juvenile separately).

Body part	*Spinaethorax spinotricosus* comb. n. male	*Spinaethorax spinotricosus* comb. n., females	*Spinaethorax spinotricosus* comb. n., juvenile
Body total	520	530–720 (605)	350–500 (445)
Head width	135	133–215 (162)	100–130 (121.2)
Head length	170	183–237 (209)	130–167 (154.8)
R1 labrum	8	9–12 (10)	7–8 (7.6)
R2 labrum	10	10–14 (11.5)	8–10 (9.3)
Antenna	120	125–145 (132.6)	94–112 (106.3)
Ant. I	10	11–13 (11.4)	9
Ant. II	23	25–30 (26.4)	18–23 (21.6)
Ant. III–IV	87	90–104 (97.5)	67–81 (76)
Ant. IV sensillum Sy	16	16–18 (16.6)	10–12 (11.3)
Ant IV macrosensilla S	23–29	20–32 (23–31)	14–24 (14.8–22.6)
Tibiotarsus I width	17	17–18 (17.5)	15–17 (16.4)
Tibiotarsus II width	17	16–19 (16.8)	14–17 (16)
Tibiotarsus III width	17	16–19 (17.4)	16–18 (16.5)
Unguis I	27	26–33 (28.7)	18–23 (21.2)
Unguis II	25	24–30 (26.3)	16–21 (19)
Unguis III	23	23–30 (25)	15–21 (18.8)
Unguiculus I	14	13–16 (14.1)	10–12 (11.2)
Unguiculus II	14	14–18 (15.5)	10–12 (11)
Unguiculus III	15	14–18 (16.1)	9–13 (11.5)
Manubrium	54	56–66 (60.3)	35–45 (42.5)
Dens (proximal part, dp)	32	29–36 (32.3)	20–26 (23.3)
Dens (distal part, dd)	56	66–83 (71.2)	41–57 (55.6)
Mucro	67	65–87 (73.6)	42–58 (53.4)
Mucro width (middle part)	6	7–9 (7.7)	5–7 (6.1)
Macroseta on oral fold	25	25–30 (28)	18–23 (21)
Spines IL_1_ on head	37	35–42 (37.3)	28–30 (28.8)
Spines IL_2_ on head	33	28–36 (31.1)	21–26 (23.9)
Spines IL_3_ on head	30	28–33 (29.8)	17–25 (22.5)
Spines IL_4_ on head	20	17–21 (18.1)	12–15 (14)

Head. Head length and width 215 and 145 µm, respectively. No eyes. Head with smooth, pointed ordinary setae and spines of different width and length (Fig. 1). Frontal part with ordinary setae (lateral ones longer than axial, 26–30 µm, respectively 10–16 µm), seta a0 present; medial part between posterior s.f. ordinary or slightly spine-like setae (20 µm); posterior part with 3+3 stouter spines IL_1_–IL_3_ of different lengths (28–42 µm) and 1+1 smaller axial spines IL_4_ (17–21 µm), others similar to smaller setae (10–14 µm). Labrum with 5,5,4 setae, 4 prelabrals. Pattern of labral setae (Fig. 2) after Massoud and Vannier (1967): a-row: 2R_1_ + 2R_2_, m-row: m + 2r_1_ + 2r_2_ and p-row with 5 ordinary setae (11 µm). Anterior R_1_ and R_2_ slightly thick, smooth and curved, R_2_ (11 µm) longer than R_1_ (9 µm). Medial setae (m-row) equal (11 µm), smooth median setae in one line with others. Maxillary palp simple, with 1 enlarged terminal seta (18 µm), 1 basal seta (14 µm) and 1 sublobal hair (Fig. 2). Basomedian field of labium with 6+6 setae (Fig. 5), median ones slightly longer (12 µm) than others (10 µm); basolateral field with 1+1 setae (10 µm), oral fold with 1+1 basal setae (8 µm) and 1+1 terminal sword-like macrosetae (25–30 µm). Head with 3+3 smooth postmedian setae ventrally (Fig. 5); 2+2 anterior setae equal (16 µm); posterior 1+1 seta slightly curved at tip (18 µm). Mandible with 4 apical teeth, medial ones longer (Fig. 3). Maxilla as in Fig. 4.

Thorax and abdomen (Fig. 6). Dorsally with ordinary setae, swollen sensilla s1, s2, s3, s3´, spines of different size (4–6 and 25–45 μm), 6+6 wax rods (wrc1, 2, 4, 6–8) with straight setae (3–4 µm) and 2+2 (wrc 3 and 5) with thicker and longer straight setae (6–7 µm). All wrc placed in small cuticular depressions. Trichobothria or their sockets not observed. Th. II with 3+3 ordinary setae (12–14 µm) and 6+6 stouter spines (25–36 µm) around thoracic sensory fields, axial spines smaller (25 µm); sensory fields at leg II base with 2+2 ordinary setae (20 µm), 3+3 stouter spines, anterior ones longer (45 µm) than posterior (26–28 µm), 1+1 lateral sensillum s1 (8 µm) broadened at tip and 1+1 swollen transparent rod with bifid tip and no base (8 µm) above s.f. of leg II. Th. III with 5+5 ordinary setae (6–8 µm), 4+4 wrc (wrc 1–4) and several small spine-like microsetae, whose overall number is not seen clearly; at leg III base with 1+1 ordinary seta (16 µm), 3+3 stouter spines, medial ones longer (42 µm) than lateral (22 µm) and 2+2 wrc (5, 6). Anterior Abd. medially with 1+1 wrc 7 and 2+2 swollen sensilla s3 and s3´; hind Abd. with abdominal sensory fields, which are surrounded by 2+2 ordinary setae (12 and 18 μm), 1+1 stouter spines (30 µm), 1+1 swollen sensilla s2 and 1+1 wrc 8 above Abd. s.f. Dorso and dorso-lateral anterior and hind abdomen covered with numerous spine-like microsetae (4–6 μm) arranged as in Figs 6 and 10. Their overall number is not seen clearly. Abd. tergum VI with 3+3 setae (17 µm) and 1 unpaired axial seta (14 µm). Anal complex with three anal valves, each with on seta (7 µm). Abd. VI sternum with 4+4 setae (18–20 µm) and 2+2 very small globular structures (1–2 µm) next to anal valve setae. Female genital plate (Abd. V sternum, Fig. 10) with 4+4 setae (8–11 µm) and 1+1 axial microsetae (4 µm). Male genital plate with 5+5 ordinary setae (12 µm) arranged in circle and with 2+2 spine-like setae (7 µm) difficult to observe (Fig. 9), laterally surrounded with 4+4 setae (18 µm). Abd. IV sternum with 3+3 setae in one row (18–20 µm), one seta more laterally and 1+1 distal setae (8 µm). Lateral part of Abd. IV sternum with 1+1 short and pointed neosminthuroid setae (6 µm) (Fig. 10).

Appendages. Ant. III and IV not separated (Fig. 11). Length of antennae 140 µm, ratio antenna/head = 0.65; length of antennal segments I, II, III–IV as 11, 26 and 103 µm. Ant. I furnished with 3 short setae (8–10 µm). Ant. II with 1 medial seta and 5 apical setae arranged in a whorl. Ant. III organ consists of 2 transparent rods (7 µm), 1 leaf-like transparent sensillum Sg (12 µm) and spine-like seta (7 µm). Proximal part of Ant. III bears 1 globular sensillum Sg (4 µm). Ant. IV with 13 curved macrosensilla S finely blunt at tip (24–32 µm); subapically with 1 long and thick subapical sensillum Sy (16–18 µm) and with 1 thick shorter apical sensillum Sx broadened at tip (12–14 µm); Ant. IV organ like a tiny, hardly visible spine (5 µm); apically with curved setae a and sa apical in position (10 µm). Complete chaetotaxy of antennae provided in Table 2. Setae numbers of legs I–III (Figs 13–15): scx I: 1, 1, 3 (one spine); scx II: 1, 1, 1; coxae: 1, 1, 2; trochantera: 4, 3, 3; femora: 8, 8, 8 and tibiotarsi: 13, 14, 13. Thin meso- or microsetae as in following numbers on leg I: femur with 2; on leg II: femur with 1; on leg III: trochanter with 1, femur with 1. For complete setation of legs see Table 3. Tibiotarsal tenent hairs ordinary, straight and pointed (16–18 µm). Unguis narrow, in distal part bended; both unguis and unguiculus unequally long in leg I, II and III: unguis 31, 27 and 26 µm, respectively, unguiculus 14, 15 and 16 µm, respectively. Length ratio unguis I, II, III / ti. I, II, III width (31, 27, 26/ 17, 16, 17 µm) = 1.8, 1.7, 1.5. Unguis I and III with three auxiliary lamellae la, lp, Bp, unguis II with only lp and Bp (Fig. 14); unguiculus I with small internal tooth on distal part, II and III untoothed and III with bended tip; unguiculi without apical filament and basal lamellae. Ventral tube with 2+2 distal setae and without posterior lobe (Fig. 7). Retinaculum with 3+3 teeth, no setae on corpus (Fig. 8). Furcula well developed (Fig. 12), length of manubrium, dens (dp and dd) and mucro: 56, 31, 71 and 74 µm, respectively. Manubrium with 4+4 setae posteriorly, lateral ones shorter (10–15 µm) than axial (16–20 µm). Dens in proximal part (dp) with 2+2 posterior setae, lateral ones (14 µm) shorter than those axial (22 µm); distal part (dd) apically with 2+2 broad, blunt lateral spines (9 µm) and 1 medial sharp spine (7 µm) on anterior side; with 2 external (E1–E2) posteriorly and 2 internal (J1–J2) spines (6–7 µm each, distal with short apical filament), and 1 medial, subapical seta (18 µm). Mucro with serrated lamellae and rounded tip. Base of mucro furnished with small scale without base. Middle mucro width 7 µm.

**Table 2.** Chaetotaxy of antennae in *Spinaethorax spinotricosus* comb. n.

	*Spinaethorax spinotricosus* comb. n.			
	Chaetae	S	Sg	Misc
Ant. I	3	0	0	
Ant. II	6	0	0	
Ant. III	15	0	0	2 Sensory organs, 1 spine-like chaeta, 1 globular sensillum Sg, 1 leaf-like sensillum Sg
Ant. IV	13	13	0	1 Sx; 1 Sy; 1 Or; 1a; 1sa

**Table 3.** Setation of legs in *Spinaethorax spinotricosus* comb. n.

	*Spinaethorax spinotricosus* comb. n.		
	Leg I	Leg II	Leg III
Subcoxae I	1	1	3
Subcoxae II	1	1	1
Coxae	1	1	2
Trochanter	4	3	3
Femur	8	8	8
Tibiotarsus	13	14	13

Sensory fields (Figs 1, 6, 14 and 15). 6+6 s.f. placed in depressions each with secretory rod (10–12 µm), i.e. blunt seta with basal part inserted on cuticle and placed in upper margin of the field. Following arrangement: (a) anterior and posterior field on head (s.f. 1, 15 × 10 and s.f. 2, 20 × 10 µm) each with secretory rod and 1 seta on margin (14–18 µm); (b) thoracic field (s.f. 3; 35 × 20 µm) with secretory rod, 3 internal spines (4 µm) arranged in triangle, 2 external marginal setae (12–14 µm) and 6 sword-like spines of different lengths (25–36 µm), 3 spines are in anterior position and 3 spines above s.f. in axial position; (c) fields at base of legs II and III (s.f. 4, 5; 20 × 15 µm) each with secretory rod, 2 internal spines (4 µm) in s.f. 4 and 1 internal spine (4 µm) in s.f. 5. S.f 4 with 2 marginal external setae (20 µm), 3 stouter sword-like spines, medial ones longer (45 µm) than lateral (26–28 µm), 1 lateral sensillum s1 broadened at tip (8 µm) and 1 swollen rod with split tip and without base (8 µm) above s.f. 4. S.f. 5 with 1 marginal external seta (16 µm), 3 stouter sword-like spines, medial ones longer (42 µm) than lateral (22 µm); (d) abdominal field (s.f. 6; 30 × 25 µm) with secretory rod, 1 internal spine (4 µm), 2 marginal ordinary setae (12 and 18 μm), 1 sword-like spine above s.f. (30 µm) and 1 swollen sensillum s2 on the margin of s.f. (6 μm). Wrc 8 is located above Abd. s.f.

Both sexes known.

#### Etymology.

The genus is named after the spine-like setae on thorax and abdomen.

#### Distribution and ecology.

*Spinaethorax spinotricosus* is currently known from two caves and is putatively spread in the cave systems of Yucatán Peninsula, mainly in places with accumulation of bat guano or other type of rich organic material.

#### Variation.

The young have only one proximal seta on dens.

### Spinaethorax tonoius

Taxon classificationAnimaliaCollembolaNeelidae

(Palacios-Vargas & Sánchez, 1999) comb. n.

#### Note.

This species, described from a cave in State of Guerrero, shares with new type species of the genus similar generic characters like presence of sword-like macrosetae on oral fold, globular sensillum on Ant. III., stouter spines on Th., fusion of Ant. III and IV, 3+3 setae around Abd. s.f., 2+2 setae on proximal part of dens and absence of E3 spine on distal part of dens. The main differences consist in setation of hind Abd. (numerous thickened macrosetae in *Spinaethorax tonoius* comb. n. vs. numerous spine-like microsetae in *Spinaethorax spinotricosus* comb. n.), in chaetotaxy of the apex of head (only one spine IL _1_ in *Spinaethorax tonoius* comb. n. vs. three spines IL _1_–IL _3_ in *Spinaethorax spinotricosus* comb. n.), in the structure of tenent hairs on Ti. (more developed in *Spinaethorax tonoius* comb. n. vs. shorter in *Spinaethorax spinotricosus* comb. n.) and in setation around Abd s.f. (absence of axial spine in *Spinaethorax tonoius* comb. n. vs. presence of spine in *Spinaethorax spinotricosus* comb. n.). Species description is based only on one adult specimen (holotype) and does not allow us to describe it in an appropriate way, along with drawings and measurements.

#### Discussion.

 The family Neelidae was for a long time an overlooked group of Collembola, mainly due to its small size and lack of diagnostic characters. In spite of the foregoing facts recent years molecular phylogenetic analysis have revealed unexpected diversity within this family (Schneider et al. 2011). Recently *Neelus* has been revised (Kováč and Papáč 2010) as well as *Megalothorax* with a redescription of the nominal species *Megalothorax minimus* (Schneider & D´Haese, 2013). Soon after additional new taxonomical characters in genus *Megalothorax* were defined (Papáč and Kováč 2013). *Spinaethorax spinotrichosus* comb. n. and *Spinaethorax tonoius* comb. n. share many specific characters. These species were included in the genus *Megalothorax* (Palacios-Vargas & Sánchez, 1999), because some features (fusion of Ant. segments III and IV, absence of E3 spine on distal dens) indicated that those specimens belonged to this genus. On the other hand, some characters link those specimens to *Neelus* (dp with 2+2 setae - except *Neelus fimbriatus*, 1+1 neosminthuroid setae at the base of Abd. IV sternite). Schneider and D´Haese (2013) stated that chaetotaxy of *Megalothorax spinotricosus* clearly differs from the other *Megalothorax* by the presence of great number of microsetae on Abd. and pointed out that *Megalothorax spinotricosus* deserved its own genus. On the basis of recent diagnostic features, material of these two species was re-examined arriving to the same conclusion as Schneider and D´Haese (2013) that they represent a new genus in the Neelidae. *Spinaethorax* gen. n. differs from other genera by striking morphological features and combinations, which clearly separate them, e.g. sword-like macrosetae on oral fold, six setae on basomedian field of labium, Ant. III and IV fused, presence of small globular sensillum Sg on Ant. III, three marginal setae around abdominal sensory field, absence of spine E3 on dd and dp with two seate. For comparison with other genera see Table 4. 

**Table 4.** Differential characters for the genera of the order Neelipleona.

Character	*Megalothorax* Willem, 1900	*Neelides* Caroli, 1912	*Neelus* Folsom,1896	*Zelandothorax* Delamare Deboutteville & Massoud, 1963	*Acanthoneelidus* Bretfeld & Griegel 2006	*Spinaethorax* gen. n.
Sensory fields	yes	no	yes	yes	yes	yes
Ant. III/IV fused	yes	no	no	yes	no	yes
Retinaculum teeth	3+3 or 4+4	2+2	3+3	4+4	4+4	3+3
Neosminthuroid chaetae at the base of Abd. IV sternite	2+2	4+4 or 5+5	1+1	2+2	1+1	1+1
Dental proximal setae (dp)	1	1	2	1	1	2
Nr. of setae on basomedian field of labium	3+3 or 4+4	2+2	4+4	-	-	6+6
E3 spine/chaeta on distal part of dens (dd)	no	yes	yes	no	no	no
Nr. of setae around Abd. sf	5	absent sf	2	-	5	3

### Identifiaction key to the World genera of Neelidae

The identification key is based on that of Bretfeld (1999).

**Table:** 

1	Head and body with well developed sensory fields; R_1_ labrum setae shorter than R_2_; labrum without apical fringes and split structures; retinaculum with 3+3 or 4+4 teeth	2
–	Large sensory fields absent; R_1_ labrum setae longer than R_2_; labrum with apical fringes and split structures; retinaculum with 2+2 teeth	*Neelides* Caroli, 1912, type species *Neelides folsomi* Caroli, 1912; Italy
2	Dens with short conical spines posteriorly; median labral setae present; a-row of labrum with 4 or 6 setae	3
–	Dens with broad triangular spines posteriorly; median labral setae missing; a-row of labrum with 5 setae	*Zelandothorax* Delamare Deboutteville & Massoud, 1963, type species *Megalothorax novozealandiae* Salmon, 1944 (New Zealand)
3	Ant. III and IV not separated	4
–	Ant. III and IV separated with suture	5
4	Sensory fields of abdomen with 5 marginal setae; 2+2 neosminthuroid setae; basomedian field of labium with 3+3 or 4+4 setae; proximal part of dens with one seta	*Megalothorax* Willem, 1900, type species *Megalothorax minimus* Willem, 1900; Belgium
–	Sensory fields of abdomen with 3 marginal setae; 1+1 neosminthuroid setae; basomedian field of labium with 6+6 setae; proximal part of dens with two setae	*Spinaethorax* gen. n., type species *Spinaethorax spinotricosus* comb. n. (Mexico)
5	Sensory fields of abdomen with 2 marginal setae; apex of head without spines; proximal part of dens with two setae (only *Neelus fimriatus* with one seta)	*Neelus* Folsom, 1896, type species *Neelus murinus* Folsom, 1896; United States of America
–	Sensory fields of abdomen with 5 marginal setae; apex of head with blunt spines; proximal part of dens with one seta	*Acanthoneelidus* Bretfeld & Griegel, 2006, type species *Acanthothorax pratensis* Bretfeld & Griegel, 1999 (Poland)

## Supplementary Material

XML Treatment for Spinaethorax

XML Treatment for Spinaethorax tonoius
